# Retinal injury from a laser skin resurfacing device during medical tourism: a public health concern

**DOI:** 10.1186/s12886-024-03383-z

**Published:** 2024-03-26

**Authors:** Lester H. Lambert, Brett L. Tompkins, Ian C. Uber, Kapil G. Kapoor, David J. Ramsey

**Affiliations:** 1 Department of Ophthalmology, Naval Medical Center Portsmouth, 620 John Paul Jones Cir, 23708 Portsmouth, VA USA; 2 Wagner Kapoor Institute, 1800 Republic Road, Suite 102, 23454 Virginia Beach, VA USA; 3 Department of Ophthalmology, Tufts University School of Medicine, 02111 Boston, MA USA; 4 Division of Ophthalmology, Lahey Hospital & Medical Center, 1 Essex Center Drive, 01960 Peabody, MA USA; 5 New England College of Optometry, Graduate Faculty, Boston, MA 02115 USA

**Keywords:** Retina, Laser injury, Chorioretinal scar, Vitreomacular traction, Neovascular membrane, Safety

## Abstract

**Background:**

Laser skin resurfacing is a popular cosmetic procedure for noninvasive skin rejuvenation. Since health insurance plans often do not cover these types of procedures, patients often pay out of pocket. Consequently, there is an incentive to go abroad, where prices are more affordable. However, practitioners in destination countries may lack rigorous training on laser safety, regulatory oversight, or licensing, especially on devices used for “cosmetic” procedures. In certain cases, this can lead to tragic outcomes, especially when underqualified practitioners operate medical-grade laser devices.

**Case presentation:**

A 29-year-old woman suffered a retinal burn from a handheld Q-switched neodymium-doped yttrium aluminum garnet (Nd:YAG) laser pulse device used to perform skin resurfacing treatment at a medical spa in Vietnam. The patient was not adequately informed about the potential risk to her vision and was not provided with any eye protection. A momentary, unintended laser exposure to the patient’s right eye led to irreversible vision loss due to a macular burn. This incident caused immediate pain, followed by the sudden appearance of floaters, along with a retinal and vitreous hemorrhage. Despite treatment with off-label bevacizumab for the development of a choroidal neovascular membrane, vision remained at the level of counting fingers because of the presence of the macular scar.

**Conclusion:**

When utilizing laser-based devices, it is crucial to employ safety measures, such as the wearing of safety goggles or the use of eye shields to protect ocular tissues from potential damage. The growing availability of cosmetic laser devices presents a substantial public health risk, because numerous operators lack adequate training in essential safety standards, or they neglect to follow them. Furthermore, patients seeking services abroad are subject to the regulatory practices of the destination country, which may not always enforce the requisite safety standards. Further research is needed to determine regional and global incidence of laser-related injuries to help direct educational and regulatory efforts.

## Background

Laser skin resurfacing, also known as light-based photo rejuvenation or intense pulsed light therapy, is a non-invasive, cosmetic procedure used for skin rejuvenation. During treatment, a handheld device emits controlled pulses of light produced by a laser or light-emitting diode (LED), which are absorbed by pigmented skin structures (e.g., melanin or hemoglobin), leading to its therapeutic effects [1, 2]. This technique is often used to address signs of aging like wrinkles, solar lentigo, and uneven skin pigmentation, or to reduce the visibility of traumatic wounds or postoperative scars [1–3]. 

While laser skin resurfacing is generally safe and effective when performed by trained professionals who adhere to appropriate safety measures, the growing popularity of light-based cosmetic procedures, coupled with the increased availability and use of unregulated devices, amplifies the risk of complications. As most health insurance plans do not cover cosmetic procedures, individuals often seek treatments outside of traditional medical settings. Some individuals even travel outside their countries of origin to obtain more affordable prices for cosmetic services [4]. Depending on local regulations, lasers in these settings may be operated without proper training or precautions to ensure patient safety [4]. While those seeking laser skin resurfacing are often counseled on the risk of dermatologic adverse effects [1], far fewer are warned that ocular tissues are vulnerable to injury by laser radiation [5, 6]. Here, we present the case of a woman who suffered a retinal burn resulting in permanent vision loss as a direct result of a laser skin resurfacing procedure at a medical spa in Vietnam.

## Case presentation

A 29-year-old woman sought laser skin resurfacing treatment in Vietnam to address uneven pigmentation. She chose this setting because the procedure was not covered by her health insurance in the United States. The procedure was performed using a handheld Q-switched neodymium-doped yttrium aluminum garnet (Nd:YAG), without appropriate pre-procedural disclosure of potential risks or the provision of any eye protection. Regrettably, during the treatment, the esthetician lost control of the handheld device, resulting in an unintentional laser emission into the patient’s right eye. This incident caused the patient to experience a flash of light followed by pain and the appearance of floaters, accompanied by an immediate loss of central vision and the development of a scotoma.

**Fig. 1:**
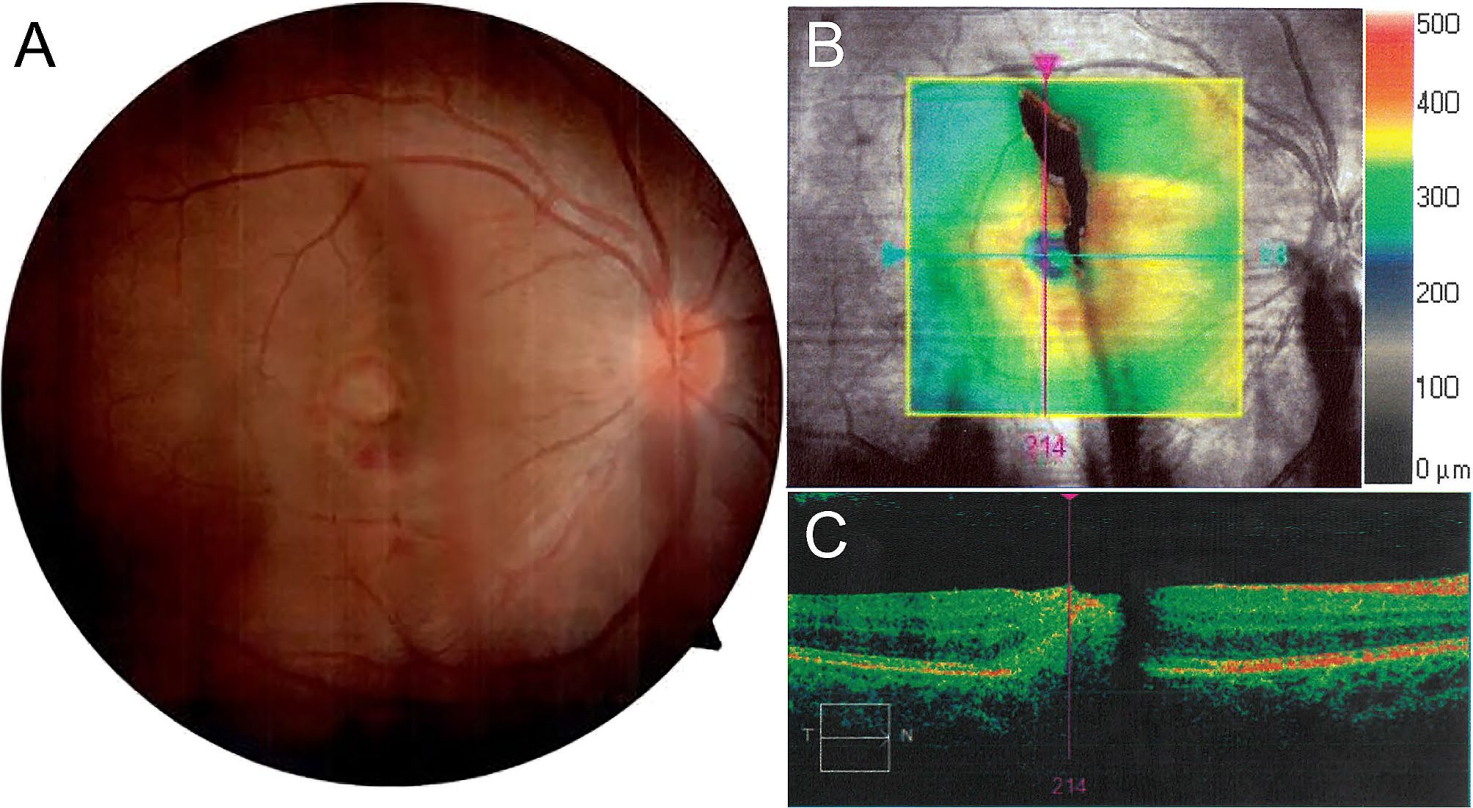
Multimodal imaging of the fundus of the right eye 11 days after a retinal injury from a handheld Q-switched Nd:YAG laser pulse device. (**A**) Color fundus photograph of the right eye shows retinal and vitreous hemorrhage in the region of the photoablative, thermal injury to the central macula. Image quality is constrained by the ocular media. (**B**) Infrared reflectance image overlaid with the en face spectral domain optical coherence tomography (SD-OCT) imaging scan area illustrating retinal thickness topography (highlighted in colored area). The green arrow shows the position of the scan line used to generate the cross-sectional OCT image of the retina. (**C**) SD-OCT cross-sectional retinal image reveals a full-thickness disruption of the retinal laminae, with a subfoveal hyperreflective lesion extending into the subretinal space (temporal to nasal: T → N). Preretinal and vitreous hemorrhage create noticeable shadows that limit scan quality

Upon return to the United States four days later, her symptoms of vision loss had not improved. She sought medical attention at a Military Treatment Facility operated by the U.S. Navy as a dependent of an active-duty military service member. Before the skin resurfacing procedure, her vision was 20/20 in both eyes without correction, having undergone laser-assisted in situ keratomileusis surgery two years prior. On initial assessment after the accident, her best corrected visual acuity was count fingers in the right eye and remained 20/20 in the left eye. External exam was unremarkable and intraocular pressures were normal. She had no afferent pupillary defect. Initial slit lamp exam revealed a vitreous hemorrhage obscuring the details of the right macula. Ultrasound through the vitreous hemorrhage confirmed that the retina was attached. Follow-up examination of the right fundus 11 days after the laser exposure identified a photoablative thermal injury to the central macula with pre-retinal hemorrhage (Fig. 1A). Optical coherence tomography (OCT) revealed the laser injury passed through the fovea, resulting in both intraretinal and subretinal hemorrhage (Fig. 1B and C). Based on these findings, the patient was counseled that her vision loss was likely permanent.

**Fig. 2:**
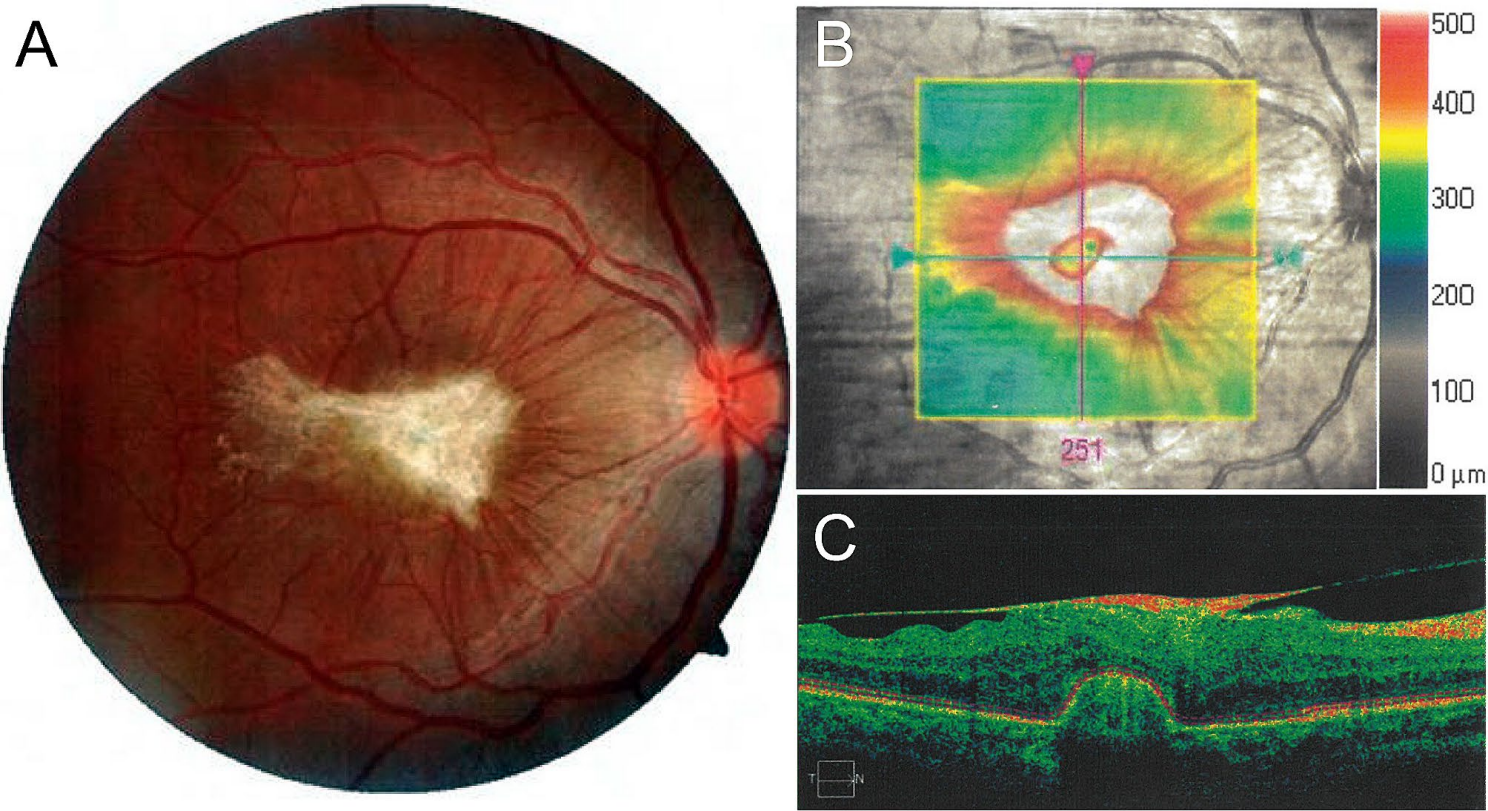
Repeat imaging of the fundus of the right eye three months after the laser injury. (**A**) Color fundus photograph of the right eye illustrates the formation of a central fibrin scar with macular traction. (**B**) Infrared reflectance image overlaid with the en face SD-OCT imaging scan area illustrating retinal thickness topography (highlighted in colored area). The green arrow shows the position of the scan line used to generate the cross-sectional retinal OCT image. (**C**) SD-OCT cross-sectional retinal image demonstrates chorioretinal scarring beneath the fovea with thickening of the adjacent retinal layers with an overlying epiretinal membrane causing vitreomacular traction (temporal to nasal: T → N)

**Fig. 3:**
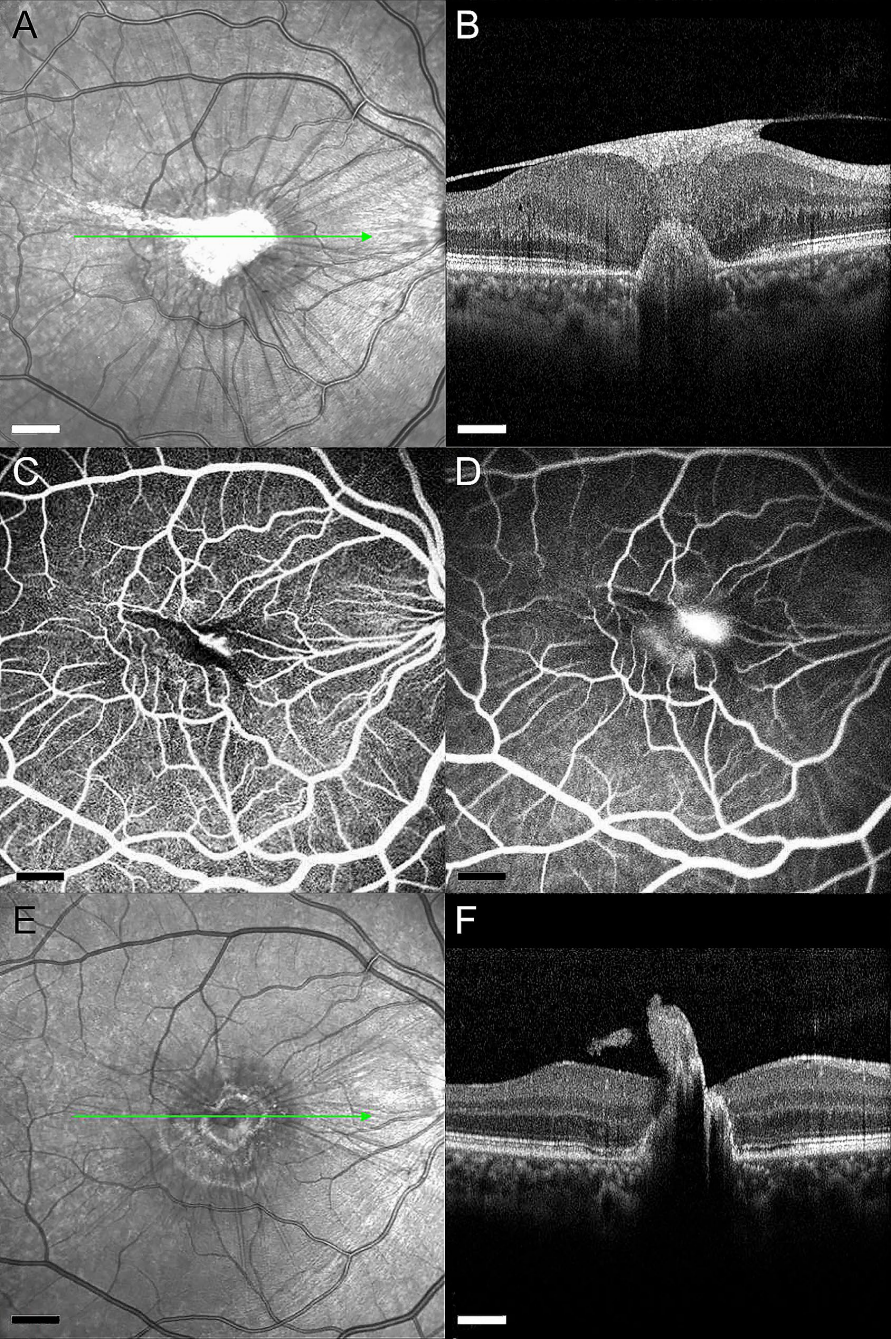
Multimodal imaging of choroidal neovascularization secondary to laser injury. (**A**) Infrared reflectance image obtained at approximately four months after the injury illustrates progression of an epiretinal membrane with vitreomacular traction. The green arrow shows the position of the scan line used to generate the cross-sectional retinal OCT image. (**B**) SD-OCT cross-sectional retinal image through the macular scar demonstrates increasing retinal thickness with reactive gliosis and intraretinal and subretinal hyperreflective material. (**C**) Fluorescein angiography of the right eye in the early arteriovenous phase demonstrates absences of retinal vessels in the region of the macular scar with adjacent hyperfluorescence corresponding to a neovascular lesion (17 s). (**D**) The fluorescence from the neovascular lesion increases during the intermediate phase (2 min 10 s), with increasing leakage in the late phases (not shown). (**E**) Infrared reflectance image obtained six months later demonstrates resolution of the fibrovascular membrane complex. The green arrow shows the position of the scan line used to generate the cross-sectional retinal OCT image. (**F**) SD-OCT cross-sectional retinal image through the macula reveals a remarkable resolution of the of the epiretinal membrane with improvement in macular contour and regression of the choroidal neovascular membrane. A tuft of scar tissue is all that remains of the fibrovascular complex. Scale bar: 500 μm

**Fig. 4:**
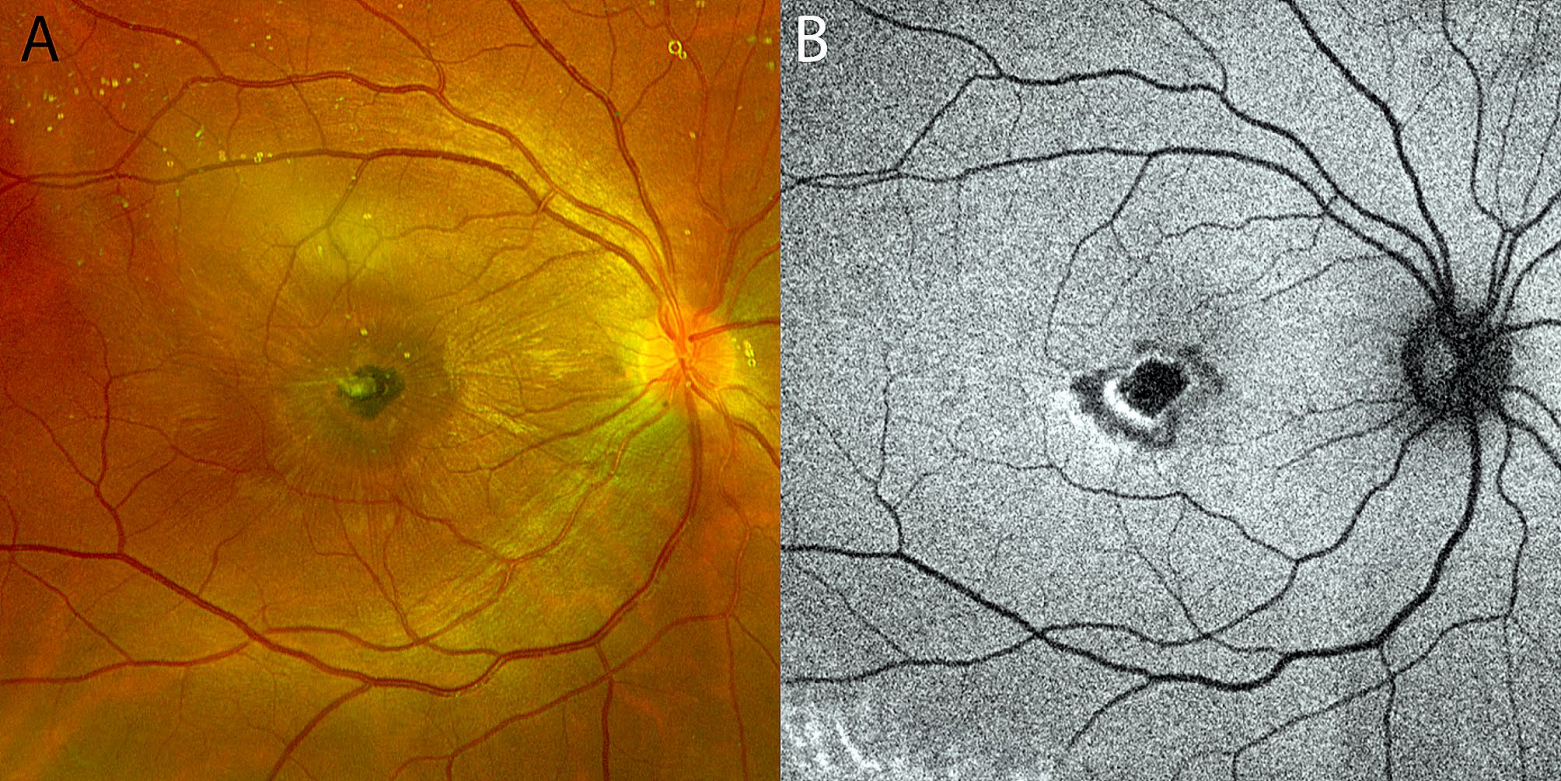
Macular scar evolution one year after a retinal injury from a handheld Q-switched Nd:YAG laser pulse device. (**A**) Color fundus photograph illustrates a well-defined macular scar which has expanded slightly beyond the site of the original injury. (**B**) Autofluorescence imaging illustrates complete loss of macular fluorescence at the site of the laser burn with collateral damage caused by the thermal injury illustrated by hyperautofluorescence surrounding the site of the laser burn

Three months later, her visual symptoms and acuity were unchanged. Examination of the fundus identified the proliferation of scar tissue in the central macula with vitreomacular traction (Fig. 2A). Repeat OCT imaging through the central macula revealed diffuse retinal thickening from an overlying epiretinal membrane with reactive gliosis and chorioretinal scarring at the site of the laser injury (Fig. 2B and C). The patient was referred to a civilian retinal specialist for further assessment and surgical evaluation. Four months after the accident, progressive epiretinal membrane formation caused distortion of the retinal contour, which is evident in OCT images displaying increased retinal thickening (Fig. 3A and B). A fluorescein angiogram identified the development of a choroidal neovascular membrane as a delayed consequence of the laser injury (Fig. 3C and D). Surgical intervention was deferred in favor of anti-vascular endothelial growth factor treatment. The patient underwent six monthly, intravitreal injections of bevacizumab (1.25 mg), resulting in the resolution of the choroidal neovascular membrane, release of the fibrovascular complex, and improvement in macular contour (Fig. 3E and F). Unfortunately, the patient’s visual acuity did not improve and remained unchanged at the level of counting fingers due to a persistent macular scar (Fig. 4A and B).

## Discussion

According to the American Society for Dermatologic Surgery, there were 4.1 million laser, light, or energy-based cosmetic procedures performed in the United States (U.S.) in 2019 [7]. The global market for skin resurfacing reached a value of 217 million U.S. dollars, and it is projected to grow at an annual rate of more than 7% over the next decade [8]. The global incidence of complications from cosmetic lasers, including cases of vision loss, remains unknown.

While retinal injuries caused by handheld laser pointers are relatively common [9], there have been fewer reported injuries from cosmetic laser devices [2, 5, 6], and no published cases have resulted from the emerging practice of medical tourism. In the U.S., all laser-based devices sold for human and veterinary use are classified, with users required to adhere to mandatory performance standards and safety regulations set by the U.S. Food and Drug Administration and the Occupational Safety and Health Administration. The handheld Q-switched Nd:YAG pulsed laser device used for skin resurfacing in this case typically operates at wavelengths of 800 or 1064 nm, with fluence ranging from 2 to 5 J/cm², which has an energy level capable of inducing retinal burns [5, 6, 9]. It would be categorized as a Class IV laser device in the U.S. and would have necessitated protective eyewear by both operators and patients to prevent laser-induced damage to ocular structures [10, 11]. The incidence of cosmetic laser ocular injury in Vietnam is unknown. Our case may represent the first case of laser injury in that nation. Although Vietnam’s Ministry of Health classifies laser devices [12], it does not include any reference to laser safety guidelines similar to those used in the United States. Further, the most recent international report on laser guidelines and regulations was published by the World Health Organization in 1982 [13]. 

Although the laser’s wavelength or parameters in the case presented are not known, the Nd:YAG pulsed laser device possessed enough photo-disruptive power to cause a blinding, full-thickness retinal burn, resulting in a retinal and vitreous hemorrhage. The initial burn also caused a breakdown of the blood-retinal barrier, leukocyte extravasation, and the release of inflammatory mediators with thrombin and fibrin deposition at the site of the injury [9, 14]. Over the course of several months, extensive glial proliferation and pre-retinal fibrosis developed driven by the secretion of growth factors, which distorted the macular contour with significant traction. Although surgical intervention was considered to remove the epiretinal membrane, the identification of choroidal neovascularization prompted the off-label use of the anti-vascular endothelial growth factor agent, bevacizumab. Several previous studies report the off-label use of bevacizumab [5], aflibercept [15], or ranibizumab [6] to manage choroidal neovascularization and associated retinal hemorrhage, which may be more aggressive and rapid in onset compared with those associated with age-related retinal diseases [6]. This management approach effectively treated the choroidal neovascularization, but, more importantly, the case presented is the first case to illustrate the utility of these agents to dramatically reverse the aggressive epiretinal scar tissue capable of deforming the macula. In rare cases, trauma-associated pre-retinal fibrosis has been reported to lead to retinal detachment [14]. 

Safety measures required when utilizing laser-based devices include the wearing of safety goggles or placing eye shields over the closed eyelids, as inadequate shielding may expose the ocular tissues to damage from unintentional exposures [16]. Symptoms of an ocular laser burn include eye pain, redness, light sensitivity, blurred vision, the sudden appearance of new floaters or a scotoma, excessive tearing, or headache [5, 6, 9]. Lack of external signs of damage may lead to an underestimation of intraocular damage, but in the case of any suspected laser-induced ocular injury, immediate referral to an ophthalmologist is indicated [16]. Recommendations regarding the use of topical, intravitreal, or high-dose systemic steroids, vitamins, or antibiotics vary among different authorities [2, 17]. However, in the majority of cases, observation alone is usually adequate for management [16]. In cases complicated by choroidal neovascular membrane formation, the intravitreal administration of drugs targeting vascular endothelial growth factor has demonstrated efficacy; however, as seen in our case, these interventions may not always lead to vision recovery [5, 6, 15]. In rare instances, surgical intervention might be warranted to address late complications, including the clearance of vitreous hemorrhage, excision of scar tissue, or repair of a macular hole or retinal detachment [9, 14]. Finally, consequences of ocular laser injuries extend beyond physical harm, potentially causing disfigurement or disability. These injuries can substantially impact various aspects of life, including career opportunities, relationships, financial stability, and mental health.

Our study is limited by virtue of being a single case report with a relatively short follow-up period. We also lack information on the incidence of laser-associated eye injuries in Vietnam or similarly developed nations. Further research is needed to establish the worldwide prevalence of ocular injuries from cosmetic laser devices. Identifying regions with high incidence rates of laser-related eye injuries would provide valuable insights for implementing laser safety education, operator training, and supporting legislative efforts to enhance regulations on laser devices [16]. Surveying ophthalmologists worldwide could generate the global incidence data needed to support timely public health interventions [18]. 

## Conclusion

In conclusion, our case highlights the potential for macular injury due to accidental exposure to Nd:YAG laser pulse devices, resulting in laser-induced maculopathy complicated by a choroidal neovascular membrane and epiretinal membrane complex formation. Both of these complications were effectively managed through off-label intravitreal administration of the anti-vascular endothelial growth factor agent bevacizumab, leading to a significant improvement in retinal anatomy and stable visual function. Although most people possess a blink reflex capable of responding in less than 0.25 s [19], without proper eye protection, even brief laser exposures can induce vision-impairing retinal damage. As more consumers seek lower-cost laser-based cosmetic services, especially in settings where safety regulations may be less stringent or lack regulatory enforcement, we may see more such eye injuries. Individuals contemplating traveling overseas for a medical procedure, such as laser skin resurfacing, should review laser safety regulations, wear appropriate eye protection, and thoroughly evaluate the practices followed by the facilities under consideration.

## Data Availability

All data generated during this study are included in this published article.

## Declarations

### Copyright statement

The authors report having no proprietary or commercial interest in any materials discussed in this letter. The views expressed in this correspondence are those of the author(s) and do not necessarily reflect the official policy or position of the Department of the Navy, Department of Defense, or the United States Government. This work was prepared as part of our official duties. Title 17 U.S.C. 105 provides that “Copyright protection under this title is not available for any work of the United States Government.” Title 17 U.S.C. 101 defines a United States Government work as a work prepared by a military service member or employee of the United States Government as part of that person’s official duties.

## Abbreviations

LED: Laser or light-emitting diode
Nd:YAG: Neodymium-doped yttrium aluminum garnet
OCT: Optical Coherence Tomography
SD-OCT: Spectral Domain Optical Coherence Tomography
